# Viable abdominal pregnancy: a case report in Yaoundé (Cameroon)

**DOI:** 10.11604/pamj.2014.18.181.4294

**Published:** 2014-06-25

**Authors:** Florent Ymele Fouelifack, Jovanny Tsuala Fouogue, Jeanne Hortence Fouedjio, Zacharie Sando

**Affiliations:** 1 Department of Obstetrics and Gynaecology, Yaounde Central Hospital, Yaoundé, Cameroon; 2 Department of Obstetrics and Gynaecology, Faculty of Medicine and Biomedical Sciences, University of Yaounde 1, Yaoundé, Cameroon; 3 Head of the Pathology Unit, Yaounde Gynaeco-Obstetric and Pediatric Hospital, Yaoundé, Cameroon

**Keywords:** Abdominal pregnancy, ectopic pregnancy, viable, Cameroon

## Abstract

We herein report a case of abdominal pregnancy managed in Yaounde (Cameroon). The 33 year old G_5_P_2022_ woman was referred to our setting for management of an abdominal pregnancy of 34 weeks diagnosed during the first routine obstetrical ultrasonography done two days earlier. This ultrasonography revealed a live foetus within intestinal loops with a severe oligoamnios. After two days of lung maturation, laparotomy was carried out and the live male baby weighed 2 600 grammes. The placenta was left on its implantation sites: omentun, uterine fundus and intestinal loops. The mother did well post-operatively and the resorption of the placenta took 11 months. The newborn presented compression deformities and died three days later of respiratory distress. This case illustrates that intra-abdominal fetuses can reach viability. Though rare, abdominal pregnancy remains a threat to mothers. Practitioners should therefore know the traps in its management.

## Introduction

Abdominal pregnancy (AP) is defined as one in which the gestational sac implants and evolves within the peritoneal cavity [1]. It is a very rare form of ectopic pregnancy accounting for 1/10 000 deliveries in developed countries [2] and between 3.4 /10 000 (Nigeria) to 1/2 256 (Congo) in sub-Saharan African countries [3–5]. Despite this important difference of incidences between poor and rich countries, AP represents almost the same proportions of ectopic all pregnancies in both settings: 1.5% (Gabon) and 1.4% (United States of America) respectively [2, 4]. In 1942 Studdiford classified AP as either primary or secondary (Studifford cited by Onan and coll.) [2]. Primary implantation on the peritoneum is extremely rare while secondary AP is more commonly reported [2, 6]. At its early stage AP is self limited by hemorrhage due to trophoblastic invasion that leads to its arrest, thus evolution beyond fetal viability is very rare [7]. The case we herein report was fortuitously discovered during ultrasonography with a viable foetus.

## Patient and observation

Mrs E.V. 33 year old, G5P2022 and married, was referred to our hospital for further management of an abdominal pregnancy of 34 weeks and 2 days. No complication was reported during antenatal care (ANC) in a community clinic by a nurse aid. History of the pregnancy revealed that she received normal prophylaxis against malaria, tetanus and anemia. Her hemoglobin level was 11.2 grammes per deciliter at 25 weeks and she was immunized against rubella and toxoplasmosis. She was negative for HIV and syphilis. No other work up was done. The first obstetrical utrasonography requested two days prior to admission revealed an abdominal pregnancy of 32 weeks and 3 days with a live foetus and severe oligoamnios. The placenta was inserted over the fundus of her polymyomatous uterus and probably on the omentum without contact with big vessels. This result prompted her referral to our service for management.

She had her first menses at 12 years old. Her menstrual cycle is regular with duration of 30 days and she bleeds for four days. She had no method of contraception and her first coïtus was at 16. She has had a total of 6 sexual partners. She reported a past history of pelvic inflammatory disease due to Chlamydia. Her blood group is A rhesus positive. She has no allergy and has never received blood transfusion. She was G_5_P_2022_. The first and third pregnancies ended by normal deliveries of two boys weighing 3 700grammes (g) and 4 300g respectively. After 8 years of secondary infertility following the first delivery she had a tubal pregnancy treated medically. The 4th pregnancy was in the right tube and led to an emergency salpingectomy following rupture. Her husband is a 33 year old smoker with tobacco index of 3.4 packet-years.

Systemic enquiry revealed painful active foetal kicks since the 16th week of pregnancy. On physical exam, her general condition was good and vital parameters were normal. Cardiac and pulmonary exams were normal. Her abdomen presented a scar corresponding to a Pfannenstiel incision. On palpation, foetal parts were easily felt just beneath the abdominal wall. The symphysio-xyphoïdal distance was 38 centimeters and the abdominal circumference was 90 centimeters. The foetus was in transverse lie and foetal heart rate was 146 beats per minute. Bowel sounds were normal. Under speculum, the cervico-vaginal mucosa was normal and the cervix was closed and deviated to the left. Digital vaginal exploration revealed a long firm and closed cervix with and increased uterus consistent with a pregnancy of 16 weeks.

**Figure 1:**
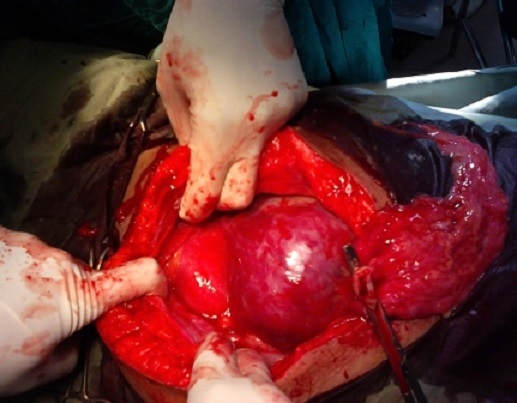
Operative findings showing the anterior part of the uterine fundus, the posterior part being hidden by the placental insertion; it also shows the omentum and the umbilical cord clamped flush with the placenta

**Figure 2:**
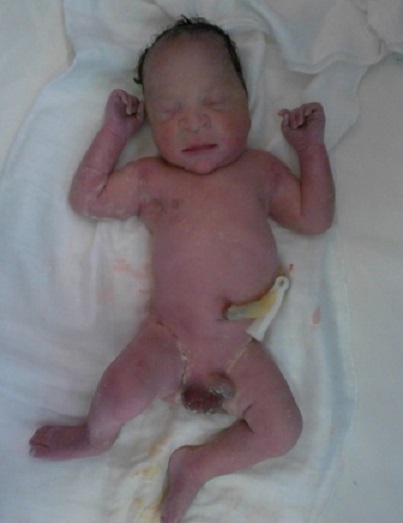
Photograph of the foetus showing the right foot with talipes valgus

Our working diagnosis was an abdominal pregnancy with a live foetus at 34 weeks and 2 days. We admitted the patient and planned a semi-urgent laparotomy after lung maturation with corticosteroids (2 intramuscular injections of 12 milligrammes of bethamethasone 24 hours apart). Anesthesiologists and digestive surgeons evaluated the patient and six pints of compatible packed red blood cells were booked. After a normal pre-operative work up, laparotomy was done under general anesthesia. The findings were: an intra-peritoneal gestational sac with severe oligoamnios containing a live male foetus weighing 2.600 g. Roughly 80% of the placenta was inserted on intestinal loops and omentun and the remaining 20% was inserted on the uterine fundus (Figure 1). The foetus was bent on the left side and his right foot was talipes valgus (Figure 2). We didn't found hemoperitoneun. The uterus was soft, globular and consistent with a pregnancy of 16 weeks. The uterine adnexae were not visible and the pelvis free of adhesions. After extraction of the foetus, the umbilical cord was cut flush with the placenta that was left in situ because of the high risk of severe bleeding.

Two Delbet corrugated drains were put in parieto-colic gutters and in the pouch of Douglas. The patient did well post - operatively and the drains were removed on the fourth post operative day. Seven days after surgery serial abdominal ultrasonography and dosages of plasmatic beta Human Chorionic Gonadotropin (β-hCG) were started. The first β-hCG level was 13 266 milliunits per milliliter and first ultrasound revealed a vascularised placental mass. The patient was discharged eight days after written counseling on danger signs that should prompt emergency consultation (abdominal pain, abdominal trauma, dizziness, extreme fatigue). The placenta and beta hCG took eleven months to disappear completely. The baby was immediately admitted in the pediatric intensive care unit where he died 3 hours later with a diagnosis of severe respiratory distress.

## Discussion

As a rare form of ectopic pregnancy AP has the same risk factors: history of infertility, ectopic pregnancy, tubal malformation, sexually transmitted infection, intrauterine device, smoking, hormonal contraception, pelvic surgery, and multiple sexual partners [2, 8]. Our patient presented with the following risk factors: history of pelvic inflammatory disease due to Chlamydia, past history of two tubal pregnancies, pelvic surgery (salpingectomy), and multiple sexual partners. Thus she was at high risk of ectopic pregnancy. Abdominal pregnancy diagnosed after the first trimester is frequent in low income countries where antenatal follow up is poor and health resources limited [4]. It is widely accepted that obstetrical ultrasonography is the cornerstone for diagnosis of abdominal pregnancy [8]. Poor access to ultrasonography may explain the high incidence observed in resource-poor setting. Our patient was poorly followed up by a nurse auxiliary who did not request for early obstetrical ultrasonography despite the very high risk of ectopic pregnancy. Despite the absence of this precious first trimester ultrasound, a skilled practitioner would have suspected AP before the painful foetal movements reported by the patient and the easiness to palpate foetal parts just beneath the skin [9].

Though some symptoms and signs are highly suggestive of AP, the diagnosis is missed in 50% of cases after clinical evaluation [5]. Other clinical presentations described include: hemoperitoneum, constipation, digestive hemorrhage, ureteral obstruction, cutaneous fistula, and failure of induction for retention of dead foetus [4]. Our patient had only severe digestive symptoms that prompted the obstetrical ultrasonography that permitted the diagnosis. Though we were not able to verify Studdiford's criteria (normal tubes and ovaries with no evidence of recent or remote injury, absence of abnormal communication between the uterus and the peritoneal cavity and the pregnancy is related solely to the peritoneal surface early enough to eliminate the possibility of secondary implantation) we can guess that our patient had a primary one [2]. Indeed, according to her past history her tubes were probably too much damaged to host and to transport an egg. Surgery is the cornerstone of management of AP and the attitude depends on the gestational age, the integrity and the localization of the gestational sac, the relationship between the placenta and the neighboring organs [9]. Before foetal viability and in absence of rupture, interruption of the pregnancy should be done by laparoscopy (if available) during the first trimester and laparotomy afterward. In case of rupture with subsequent hemoperitoneum, emergency surgery to achieve hemostasis is the recommended attitude. If the foetus is viable laparotomy should be planned after lung maturation with corticosteroids if necessary and the baby should be admitted in intensive care unit [9].

Foetal mortality is very high with a rate varying from 75 to 95% (Renaud cited by Picaud and coll.) [4]. Foetal morbidity is dominated by prematurity, hypotrophy and deformations, the later accounting for up to 20-40% (Tromans cited by Picaud and coll.) [4]. Respiratory distress syndrome is also frequent and is explained by the severe oligoamnios and the inhibition of surfactant production by hypothermia due the abdominal location (Rabarijaona cited by Guèye and coll.) [9]. In our case there was a severe oligo-amnios and the foetus died in a context of respiratory distress. There is a consensus to leave placenta in situ if its removal present any risk of bleeding. It undergoes degeneration during several weeks with a risk of infection that together with hemorrhage constitute the most dangerous maternal complications [9]. This degeneration that can be spontaneous or enhanced with anti-mitotic drugs is classically followed up by serial abdominal ultrasonography and serial dosage of plasmatic β-hCG [9, 10]. Opponents to methotrexate adjunction argue that since it is mainly active on proliferative trophoblastic cells, it carries more risks (in terms of side effects). Moreover, the bigger the mass of dead trophoblastic tissue, the higher is the risk of infection [10]. Our patient did well post operatively and was discharge under surveillance of spontaneous placenta degeneration that led to its complete disappearance.

## Conclusion

This case illustrates that though rare, AP is a serious condition that requires high level care. It is therefore important to prevent it by providing universal access to good antenatal care. Bearing it in mind is helpful to carry out correct diagnosis before specific signs for practitioners in resource-poor setting where incidence remains very high.
